# Immune Response and Evasion Mechanisms of *Plasmodium falciparum* Parasites

**DOI:** 10.1155/2018/6529681

**Published:** 2018-03-25

**Authors:** Esmael Besufikad Belachew

**Affiliations:** Biology Department, College of Natural Science, Mizan Tepi University, Tepi, Ethiopia

## Abstract

Malaria causes approximately 212 million cases and 429 thousand deaths annually. *Plasmodium falciparum* is responsible for the vast majority of deaths (99%) than others. The virulence of *P. falciparum* is mostly associated with immune response-evading ability. It has different mechanisms to evade both *Anopheles* mosquito and human host immune responses. Immune-evading mechanisms in mosquito depend mainly on the Pfs47 gene that inhibits Janus kinase-mediated activation. Host complement factor also protects human complement immune attack of extracellular gametes in *Anopheles* mosquito midgut. In the human host, evasion largely results from antigenic variation, polymorphism, and sequestration. They also induce Kupffer cell apoptosis at the preerythrocytic stage and interfere with phagocytic functions of macrophage by hemozoin in the erythrocytic stage. Lack of major histocompatibility complex-I molecule expression on the surface red blood cells also avoids recognition by CD8^+^ T cells. Complement proteins could allow for the entry of parasite into the red blood cell. Intracellular survival also assists the escape of malarial parasite. Invading, evading, and immune response mechanisms both in malaria vector and human host are critical to design appropriate vaccine. As a result, the receptors and ligands involved in different stages of malaria parasites should be elucidated.

## 1. Introduction

Malaria is one of the most important infectious diseases in the world, and it has been recognized as the most widespread infection in tropical and subtropical areas with high rate of morbidity and mortality [1]. An estimated 3.3 billion people are at risk of malaria in the world, of which 1.2 billion are at high risk and 97 countries had ongoing malaria transmission [1, 2]. It still remains a challenge in the world in general and in Africa in particular. There is an estimated 212 million cases all over the world; most of these cases (82%) were in the WHO African Regions, followed by the WHO Southeast Asia Regions (12%) and the WHO Eastern Mediterranean Regions (5%). Globally, malaria deaths were 429,000, and 90% of these deaths were in the WHO African Regions, followed by the WHO Southeast Asia Regions (7%) and the WHO Eastern Mediterranean Regions.

*Plasmodium falciparum* is mainly responsible for the enormous deaths (99%) than other plasmodium species [2, 3]. Some studies associate the virulence of *P. falciparum* with its ability to escape the human and vector immune system by different mechanisms [2]. This review is mainly focused on evading, invading, and immune response mechanisms involved in different stages of malaria parasite and on its implication for vaccine development.

The life cycle of any pathogenic microorganism is vital and must be discussed before everything. Hence, this review first highlights the life cycle of *P. falciparum*, which occurs both in female *Anopheles* mosquito vector and human host.

In Anopheles mosquito vectors, the cycle begins when gametocytes are ingested by mosquitoes, but approximately 62% of mosquitoes that ingest infected blood never get infected. Inside the mosquito's gut, gametocytes are differentiated into gametes and undergo fertilization to form zygotes. The zygotes are then differentiated into ookinetes and develop into oocyst. Out of the thousand gametocytes, only 50–100 differentiated into ookinetes, and only around five develop into oocysts. The ookinetes then invade the midgut epithelium by using mosquito sugars and parasite lectins. On the other hand, oocysts developed and produce thousands of sporozoites which invade the salivary glands. During blood meal, an infected mosquito releases sporozoites in human [4, 5].

Sporozoites then invade hepatocytes by using cholesterol uptake pathway [6]. In the hepatocyte, sporozoites are enclosed in a parasitophorous vacuole to avoid lysosomal degradation. Merozoites ranging from 10,000 to 30,000 exit the schizont within 60 seconds of hepatocyte invasion by sporozoites [7].

Merozoite surface protein (MSP) 1 plays a main role for merozoite attachment to erythrocytes. Then, reorientation mediated by merozoite apical membrane antigen (AMA) 1 results in juxtaposition of the merozoite's apical end with the erythrocyte membrane that allows closer interaction. Tight junction between the parasite and host membrane is formed to enter the RBC. Erythrocyte-binding ligands (EBLs) and reticulocyte-binding-like protein homologs (RBLs) are involved in junction formation. EBLs are encoded by EBA175, EBA140, and EBA181genes. PfRh1, PfRh2a, PfRh2b, and PfRh4 ligands are encoded by RBL genes, but the erythrocyte's receptors to these ligands are still puzzling [8]. Dense granules, micronemes, and rhoptries are considered as principal invasive organelles found in merozoites [6, 9]. In erythrocytic schizogony, a single merozoite grows and forms 16–32 daughter merozoites. The underlying cause of increased pressure and destruction of the cytoskeleton preceding rupture of erythrocytic merozoite to begin the erythrocytic cycle is not completely clear. In parallel, stress factors such as lack of nutrition and host immune pressure force merozoites to develop into gametocytes. Finally, the cycle continues when *Anopheles* mosquito ingests blood containing gametocytes [1].

## 2. Immune Response to *P. falciparum* Malaria

Immune response against the *P. falciparum* malaria parasite is multifaceted and stage specific both in *Anopheles* mosquito vector and human host. Immunological responses could also contribute to the pathophysiology of the disease in human [10]. The detailed explanations of cells and molecules involved in vector and host immune response are briefly explained in the next topics.

### 2.1. *Anopheles* Mosquito's Immune Response

*Plasmodium falciparum* malaria parasite is limited by several bottlenecks before establishing infection in its *Anopheles* mosquito vector. This includes the physical, microbiological, and immunological defenses of the mosquito immune system. Immunological defense plays a key role when ookinete traverses the midgut and sporozoites migrate to the salivary glands [11]. The interaction of the mosquito immune system is critical to control its vectorial capacity [12, 13].

#### 2.1.1. Physical Barriers

It is the first line of defense of *Anopheles* mosquito to *P. falciparum* parasites. The major physical barriers are peritrophic membrane (PM) of the midgut, cuticle of the exoskeleton, and lining of the tracheal respiratory system [12]. Capsule formation around the parasite by mosquito melanin also has a protective role [13].

#### 2.1.2. Midgut Microbiota

The microbiota found in *Anopheles* mosquitoes, such as *Asaia*, *Enterobacter*, *Pseudomonas*, and *Pantoea*, induces AMPs, which stimulate a basal innate immune activity against *P. falciparum* infection [14]. The study by Dong et al. [15] also reported an increased susceptibility to *Plasmodium* malaria infection in microbe-free mosquitoes.

#### 2.1.3. Humoral Immune Response

Complement-like or thioester-containing protein (TEP) 1 that circulates in the *Anopheles* mosquito hemolymph is the major arm of defense in the humoral immune response of *Anopheles* mosquito [14]. It forms leucine-rich repeat protein 1 (LRIM1)/anopheles plasmodium-responsive leucine-rich repeat protein (APL) 1/TEP1cut complex and gets accumulated on the ookinete surface for killing. Apolipophorin and apolipoprotein D precursors and fibrinogen-related proteins are also documented as players in midgut antiplasmodial defense [14].

A study conducted in Portugal showed that hemozoin activates transcription of several key immune genes like REL2-F transcription factor [16] that regulates TEP1, APL1, LRRD7, and FBN9 anti-*Plasmodium* immune factors [17]. Anti-*Plasmodium* response of *An. gambiae* aga-miR-305 is also demonstrated [18]. Antibodies also avert ookinete motility, penetration of the midgut wall, and formation of oocyst [19].

#### 2.1.4. Cellular Immune Response

The primary immune cells involved in mosquito innate immune response are hemocytes [20]. Hemocytes include granulocytes, oenocytoids, and prohemocyte subtypes that are involved in phagocytosis, melanization, and hematopoietic progenitors, respectively [14]. The study by Smith et al. [21] indicated LL3 influences oocyst survival and hemocyte differentiation. Other immune effectors released by hemocytes and fat body into hemolymph are also involved in phagocytosis, secretion of antimicrobial peptides, nodule formation, agglutination, encapsulation, and melanization [12]. Reactive oxygen species (ROS) produced by hemocyte is also involved in mosquito immunity against *P. falciparum* [22].

#### 2.1.5. Signaling Pathways in Mosquito Antiplasmodial Immunity

This pathway includes Toll, immune deficiency (Imd), Janus kinase (JNK), and signal transducers and activators of transcription (STAT), which contribute to anti-*Plasmodium* defense. The Toll and Imd pathways target the ookinete stage of the parasite and promote activation of the mosquito TEP1 complement-like system [13]. These pathways are activated when they recognize PAMPs, which activate NF-*κ*B that leads activation of Rel1 and Rel2 in Toll and Imd pathways, respectively. Activation of both pathways is also important for the entry of AMPs to nucleus such as defensins, cecropins, attacin, and gambicin, which have antiplasmodial activity [14]. Rel1 and Rel2 are negatively controlled by Cactus and Caspar, respectively. Potent anti-*Plasmodium* effectors such as TEP1, APL1, LRRD7, and FBN9 are also controlled by the Imd pathway. Immune-enhanced *Anopheles stephensi* mosquitoes by Rel2 in the midgut demonstrated better resistance to *Plasmodium* infection and may give clear direction to design appropriate control strategies [14, 15]. Currently, activation of the *Imd* and *Toll* pathways to induce the expression of AgDscam isoforms that have species-specific antiplasmodial responses is indicated. The genes that mediate these pathways and tissues in which they are produced need to be illustrated [13].

Janus kinase–STAT pathway is also linked with anti-*Plasmodium* defense but activation of these pathways is not clearly explained [14]. The JNK pathway also regulates the expression of HPX2, NOX5, and TEP1 in hemocytes that promote TEP1-mediated lysis. The STAT pathway targets after parasites cross the midgut and change into the oocysts [13].

Signal transducers and activators of transcription genes (STAT1/AgSTAT-B and STAT2/AgSTAT-A) mediate immunity against the *P. falciparum* malaria parasite. AgSTAT-A is involved in transcriptional activation of NO synthase that increases reactive NO and transcription of suppressors of cytokine signaling (SOCS), which lessen the development of parasite. The exact role of AgSTAT-B is not characterized [14].

### 2.2. Immune Response to *P. falciparum* in Human

The response is complex and targets at different stages of plasmodium parasites. Immune attack involvement is high in the erythrocytic stage in contrast to preerythrocytic stage, and major immune players in the preerythrocytic and erythrocytic stages are CD8^+^ T cells and antibodies, respectively [23].

#### 2.2.1. Skin as Physical Barrier

The skin is the first critical physical barrier and acts as first line of defense against many pathogens and is also true for *P. falciparum* malaria parasites. After inoculation, sporozoites stay in the skin for several hours and are activated into a state of readiness for the hepatic stages. Antibodies found in the skin tissues also inhibit sporozoite motility in the dermis [18]. Approximately 50% of the sporozoites do not leave the inoculation site [24]. As a result, this early stage could play a key role in vaccine design [16].

The sporozoite proteins (SPECT1 and SPECT2) were reported to be necessary to pass the skin barrier, cell traversal, and migration to the liver [25]. This allows for sporozoites to evade destruction by phagocytes, and growth is arrested in nonphagocytic cells in the host dermis [7].

#### 2.2.2. Immune Response to Preerythrocytic-Stage Parasites

Immune response at the preerythrocytic stage is targeted on free sporozoites and infected hepatocytes. Antibodies against free sporozoites and circumsporozoite protein (CSP) are important to prevent invasion of hepatocytes by neutralizing proteins required for cell traversal and invasion. It also activates complement fixation, phagocytosis, and lysis by cytotoxic NK and NKT cells. It also recognizes parasite neoantigens at the surface of infected hepatocytes and kills through an antibody-dependent cell-mediated mechanism by Kupffer cells and NK cells [8].

CD8^+^ T cells producing interferon-*γ* are mainly involved in killing of intrahepatic parasites. Other cells like NK, NKT, and *γδ*T cells also kill intrahepatic parasites through secretion of type I interferons and IFN-*γ* [1, 26, 27].

Unlike viruses and bacteria, *P. falciparum* malaria parasites can trigger type I IFNs in the absence of Toll-like receptors (TLR3 and TLR4) and their signaling proteins (MyD88 and TRIF); rather, they use melanoma differentiation-associated gene 5 protein (MDA5) and signaling via the mitochondrial antiviral signaling protein (MAVS), which activates the transcription factors IRF3 and IRF7 [28]. Very recently, an exoerythrocyte-form (EEF) RNA was also reported to be recognized by MDA5 in hepatocytes, triggered a type I IFN response in the innate immune cells. Host iron regulatory hormone hepcidin, which impairs the growth of sporozoites, is also produced by unknown mechanisms [7].

Killing of infected hepatocytes and blocking of invasion by CD8^+^ T cells and antibodies, respectively, are bottleneck phases that could be targeted by vaccine [29].

#### 2.2.3. Immune Response to Erythrocytic Stage of Infection

Adaptive immunity against erythrocytic-stage *P. falciparum* is more complex than other stages [19]. The release of merozoites from hepatocytes to invade RBCs is responsible for initiation of the erythrocytic stage. At this stage, the targets are free merozoites and intraerythrocytic parasites (schizonts). Humoral or antibody and T cell responses are important to control merozoites and intraerythrocytic parasites, respectively. Antibodies can opsonise merozoites for uptake or to inhibit invasion of RBCs. Antibody mediates cellular killing, blocks adhesion of infected RBCs to endothelium, and neutralizes parasite toxins to prevent the induction of excessive inflammation. It also marks merozoites for lysis by complement [25]. This stage is also known by proinflammatory cytokine response that activates macrophages [1].

The role of CD8^+^ T cells in the erythrocytic stage is negligible [19]. CD4^+^ T helper cells are also important to produce proinflammatory cytokines that activate macrophages. They also mediate an activation of specific B cell clones [30].

Others like NK cells and *γδ*T cells are also involved in the immune response [25]. IFN-*γ*, perforin, and granzyme produced by NK cells are responsible to kill *P. falciparum*-infected RBCs [31].

#### 2.2.4. Immune Response against Gametocyte

Antibodies kill gametocyte through complement-mediated lysis and prevent sequestration and maturation of gametocytes in the host. Antibodies derived from host during blood meal are also highly responsible for complement-mediated killing of gametocytes and prevent gamete fusion in mosquito. Nitric oxide produced by macrophages is also important to kill gametocytes [19].

## 3. Immune Evasion

### 3.1. Immune Evasion Mechanisms of *P. falciparumin* in *Anopheles* Mosquito

Immune evasion is a strategy used to avoid immune response attack. Similarly, *P. falciparum* parasites evade mosquito immune response to transmit to a new host. The main and critical *P. falciparum* gene used for evasion of *Anopheles* mosquito immune response is Pfs47. It inhibits JNK-mediated apoptosis by preventing activation of several caspases [32, 33]. Moreover, being deficient in caspase-S2 also prevents protein nitration in the mosquito midgut cells. Pfs47 gene also inhibits NOX5 and HPX2 [1, 34, 35].

Host complement factors (FH) have also an influence on mosquito midgut stages. The FH receptor in *P. falciparum* gamete is glideosome-associated protein 50 (GAP50) that protects the gametes from complement attack [1]. Blocking of this receptor may also shed light for vaccine development. Immune-modulatory peroxidase (IMPer) is also crucial to form dityrosine network, which helps parasites to inactivate NOS [13].

### 3.2. Immune Evasion in Human

#### 3.2.1. Immune Evasion Strategies of Liver Stage *P. falciparum* Malaria Parasites

Free sporozoites and intrahepatic parasites must pass the hurdle of host immune response in order to enter the erythrocytic stage [25]. Research showed that sporozoites actively pass through Kupffer cells (KCs, 24%) and endothelial cells (ECs, 53%); some sporozoites can cross the gaps between an EC and a KC [7], but it is puzzling how sporozoites safely pass through KCs, which kill other microorganisms [25]. To pass this barrier, the CSP binds to KC surface proteins, and this interaction produces high levels of intracellular cAMP/EPAC that prevents the formation of ROS. Sporozoites' contact with KC also downregulates the inflammatory Th1 cytokines and upregulates the anti-inflammatory Th2 cytokines [36]. In some cases, the binding of sporozoites also induced KC apoptosis and reduced the expression of major histocompatibility complex (MHC)-I. This results in induction of T cell tolerance [37]. The CSP antigen of sporozoites could also be responsible for the reduction of KC MHC-I expression [8]. Sporozoites are able to manipulate the KC functions [25].

Once inside the hepatocyte, parasitophorous vacuole prevents lysosomal degradation. Host heme oxygenase-1 (HO-1) also enhances the development of intrahepatic parasites by modulating the host inflammatory response. Sporozoite also interferes with the mTOR pathway [7].

#### 3.2.2. Intraerythrocytic Immune Evasion

The success of evasion depends on merozoites and infected red blood cell (iRBC) surface proteins. Mostly, immune evasion by intraerythrocytic parasites is the result of antigenic diversity and sequestration [1]. Intracellular survival also assists the parasite escape by avoiding direct interaction with the immune cells. Lack of MHC-I molecule expression on the surface RBCs also helps parasite to void recognition by CD8^+^ T cells. They also create rosettes that help them to bind on RBC epitopes and avoid immune recognition [25, 38].

Expression of variable antigenic surface proteins on iRBCs helps them to evade host immune response [25]. Antigenic diversity is mostly developed from multicopy gene families and polymorphic alleles. PfEMP1 is one of the highly polymorphic proteins, encoded by approximately 60 copies of *var* genes. It has different variable domains that establish their binding to various ligands on endothelial cells [1, 19]. A particular var gene, known as var2csa mediates cytoadherence of iRBC to syncytiotrophoblasts of the placenta [8, 19].

The variant proteins, such as RIFINs at early trophozoite stage and STEVORS at mature trophozoite stage [8], are possibly important in immune evasion [1]. In general, antigenic polymorphism is the most difficult hurdle for development of effective *P. falciparum* malaria vaccines [39].

The second immune evasion mechanism at this stage is sequestration, mediated by PfEMP-1, RIFIN, and STEVOR multigene families. These allow iRBC adherence to vascular endothelium that protects from clearance of the parasites by spleen [38]. Endothelium receptors such as EPCR, CSA, CD36, and ICAMs are also important for sequestration. Rosette formation and adherence are important for immune evasion through sequestration and responsible for the occurrence of cerebral malaria [25].

IgM, which is not specific for these parasites, also binds to PfEMP-1 molecules through their Fc portion (Fc) and promotes rosetting that may facilitate sequestration by preventing splenic elimination [19].

Phagocytic functions of macrophages are also hindered by *P. falciparum* malaria pigment or hemozoin. Macrophages that have hemozoin cannot phagocytose more iRBC and reduce the production of radical oxygen intermediates. *P. falciparum* infection also activates checkpoint inhibitor molecules [19].

Most *P. falciparum* parasites use sialic acid- (SA-) independent pathway to evade antibody immune response [8]. They also divert antibody response for one antigen to another antigen [19]. Polymorphic tandem repeats in antigens also mask the critical epitopes [38].

#### 3.2.3. Immune Evasion by Merozoites

The mechanisms used for merozoite evasion include antigenic proteins. Principally, merozoite surface proteins (MSPs), PfAMA1, PfEBA, and PfRHs are involved in merozoite evasion. Among them, MSPs are highly polymorphic and play a key role to evade immune attack [1]. Merozoite proteins show strong homology to the host protein that makes it difficult to be recognized by antibody [19].

Expression of RIFINs, STEVORs, and SURFINs is important for evasion but latter two remain to be elucidated [1]. Free merozoites also bind to FH and fH-like protein 1 to inactivate C3b for protection of lysis. The transmembrane protein Pf92 is a FH-binding receptor that may have a role in *P. falciparum* vaccine development [1, 19]. Complement proteins may allow the entry of parasite [38].

#### 3.2.4. Evading Mechanisms of Gametocytes

Antibodies are also enhancing transmission to the mosquito [19]. Expression of Var, Rif, and Stevor proteins that provides an immune evasion is also indicated recently [8].

## 4. Conclusions

This review manly focused on the evasion, invasion, and immune response mechanisms of *P. falciparum* involved both in *Anopheles* mosquito vector and human host. Based on this, molecular and cellular immune molecules and cells are discussed. The receptors and ligands involved in each stage are also elucidated that gives an indication for vaccine development.

## Data Availability

Data will not be shared, because it is indicated in the references.

## Abbreviations

AMA: Apical membrane antigen
APL: *Anopheles* plasmodium-responsive leucine-rich repeat protein
CSA: Circumsporozoite protein
EBA: Erythrocyte-binding antigen
EBL: Erythrocyte-binding ligand
ECs: Endothelial cells
EEF: Exoerythrocyte form
Imd: Immune deficiency
iRBCs: Infected red blood cells
JNK: Janus kinase
KC: Kupffer cells
LRIM1: Leucine-rich repeat protein 1
MHC: Major histocompatibility complex
MSPs: Merozoite surface proteins
RBL: Reticulocyte binding-like
REL: Relish
ROS: Reactive oxygen species
STAT: Signal transducers and activators of transcription
TEP: Thioester-containing protein
TLR: Toll-like receptors.
