# Correction to “Immune Response and Evasion Mechanisms of *Plasmodium falciparum* Parasites”

**DOI:** 10.1155/jimr/9816294

**Published:** 2025-12-18

E. B. Belachew, “Immune Response and Evasion Mechanisms of *Plasmodium falciparum* Parasites,” *Journal of Immunology Research*, 2018, 6529681, https://doi.org/10.1155/2018/6529681.

In the article, the source cited as Reference 16 in the following sentence of Section 2.1.3 (Humoral Immune Response) is incorrect:

“A study conducted in Portugal showed that hemozoin activates transcription of several key immune genes like REL2‐F transcription factor [16] that regulates TEP1, APL1, LRRD7, and FBN9 anti‐Plasmodium immune factors [17].”

The above section should be corrected as follows and the references list should be updated to include Reference 40 [1]:

“A study conducted in Portugal showed that hemozoin activates transcription of several key immune genes like REL2‐F transcription factor [40] that regulates TEP1, APL1, LRRD7, and FBN9 anti‐Plasmodium immune factors [17].”

We apologize for this error.
